# Downregulation of Nuclear Protein H2B Induces Salicylic Acid Mediated Defense Against PVX Infection in *Nicotiana benthamiana*

**DOI:** 10.3389/fmicb.2019.01000

**Published:** 2019-05-08

**Authors:** Xue Yang, Yuwen Lu, Xing Zhao, Liangliang Jiang, Shengchun Xu, Jiejun Peng, Hongying Zheng, Lin Lin, Yuanhua Wu, Stuart MacFarlane, Jianping Chen, Fei Yan

**Affiliations:** ^1^Department of Plant Protection, Shenyang Agricultural University, Shenyang, China; ^2^Institute of Plant Virology, Ningbo University, Ningbo, China; ^3^State Key Laboratory Breeding Base for Sustainable Control of Pest and Disease – Key Laboratory of Biotechnology in Plant Protection, Zhejiang Academy of Agricultural Sciences, Hangzhou, China; ^4^College of Plant Protection, Nanjing Agricultural University, Nanjing, China; ^5^Central Laboratory of Zhejiang Academy of Agricultural Sciences, Hangzhou, China; ^6^Cell and Molecular Sciences Group, The James Hutton Institute, Dundee, United Kingdom

**Keywords:** H2B, potato virus X resistance, salicylic acid, VIGS, RNA silencing

## Abstract

Histone H2B protein is not only structurally important for chromosomal DNA packaging but is also involved in the regulation of gene expression, including the immune response of plants against pathogens. In this study, we show that the potato virus X (PVX) infection resulted in the reduced expression of H2B at both the mRNA and protein level in *Nicotiana benthamiana*. Tobacco rattle virus (TRV)-based virus-induced gene silencing (VIGS) was then used to down-regulate the expression of H2B in *N. benthamiana* and tests showed that the titre of TRV was similar in these plants to that in control treated plants. When these H2B-silenced plants were inoculated with PVX, the virus spread more slowly through the plant and there was a lower titre of PVX compared to non-silenced plants. Abnormal leaf development and stem necrosis were observed in the H2B-silenced plants, which were alleviated in H2B-silenced *NahG* transgenic plants suggesting the involvement of salicylic acid (SA) in the production of these symptoms. Indeed, quantitative reverse transcription (qRT)-PCR and liquid chromatography tandem mass spectroscopy (LC-MS) results showed that endogenous SA is increased in H2B-silenced *N. benthamiana*. Thus, downregulation of H2B induced the accumulation of endogenous SA, which was correlated with stem necrosis and a decreased accumulation of PVX in *N. benthamiana*.

## Introduction

Histones are nuclear proteins that are classified into five major protein groups: histones H2A, H2B, H3, and H4 are known as the core histones, while histones H1/H5 are known as the linker histones (Luger et al., 1997; Bellaïche and Grégoire, 2006). The core histone octamers (two molecules of each protein) act as spools that package eukaryotic chromosomal DNA into structural units called nucleosomes.

In addition to their role in organizing eukaryotic DNA, post-translationally modified H2B proteins can modulate the nucleosome/chromatin structure or DNA accessibility to affect the transcriptional pathways linked to embryonic development and cell differentiation (Zhang, 2003; Shilatifard, 2006; Fleming et al., 2008; Kyriss et al., 2010; Ríos-Uzeda and Wallace, 2014; Rønningen et al., 2015; Sadakierska-Chudy and Filip, 2015). Histones, including H2B, are subject to a range of post-translational modifications including acetylation and ubiquitination. In plants, the development and differentiation of *Arabidopsis thaliana* stem cells correlates with changes in histone H2B acetylation (Rosa et al., 2014). In rice (*Oryza sativa*), histone H2B monoubiquitination (H2Bub1) contributes to the regulation of flowering time and yield potential (Du et al., 2016). More recently, studies have revealed that post-translational modifications of H2B are involved in plant pathogen defense and non-host resistance (Isaac et al., 2009; Conn et al., 2011; Seo et al., 2011; Ding and Wang, 2015; Ramirez-Prado et al., 2018). For example, H2B mono-ubiquitination is catalyzed by two RING E3 ubiquitin ligase enzymes, HUB1 and HUB2, and HUB1 has been shown to be necessary for resistance to various necrotrophic fungal pathogens, by regulating ET- and SA-mediated responses in Arabidopsis (Dhawan et al., 2009). Moreover, disrupting tomato H2B mono-ubiquitination by silencing *SIHUB1* and *SIHUB2* increases susceptibility to *Botrytis cinerea*, which is related to the balance between salicylic acid (SA)- and jasmonic acid (JA)/ethylene (ET)-mediated signaling pathways (Zhang et al., 2015).

Plant viruses are important plant pathogens, which cause significant agricultural losses world-wide. There are at least two major mechanisms for plants to fight against virus infection. The primary plant defense is thought to be based on RNA silencing, an evolutionarily conserved, sequence-specific, mechanism that targets invasive nucleic acids for enzymatic degradation (Voinnet, 2001; Ding and Voinnet, 2007). In Arabidopsis, four Dicer-like ribonucleases (DCLs), 10 Argonaute proteins (AGOs), five dsRNA-binding proteins (DRBs), and six RNA-directed RNA polymerases (RDRs) have been identified, which participate in at least four different endogenous RNA silencing pathways, to combat virus infection as well as ensuring spatial and temporal regulation of the gene expression throughout the plant life cycle (Qu et al., 2005; Brodersen and Voinnet, 2006; Qu et al., 2008). The second defensive system that contributes to plant defense against viral pathogens is known as plant innate immunity. A well-studied example of this is the involvement of the tobacco N protein in conferring resistance to the tobacco mosaic virus (TMV) by recognizing the virus replicase protein and leading to the initiation of local programmed cell death (PCD) and a priming of resistance in more distant (systemic) regions of the plant (Caplan et al., 2008, 2009). It is well known that phytohormones, particularly salicylic acid (SA) and jasmonic acid (JA), participate in plant innate immunity to viruses. Foliar application of JA followed by SA triggers a strong systemic resistance to TMV (Zhu et al., 2014). Furthermore, exogenous SA is an inducer of systemic acquired resistance (SAR) to the potato virus X (PVX) infection in *Lycopersicum esculentum* and *Solanum tuberosum* (Sanchez et al., 2010; Falcioni et al., 2013). However, SA-induced resistance to viruses does not involve factors such as pathogenesis-related (PR) proteins or NPR1 (non-expressor of PR1) that are involved in plant defense to bacteria and fungi (Carr et al., 2010). There is also increasing evidence that phytohormone expression and activity is closely integrated with the activity of some components of the RNA silencing system in plants (Yang et al., 2004; Campos et al., 2014; Alazem et al., 2017).

In this study, we examined H2B accumulation in PVX-infected *Nicotiana benthamiana* plants and found that PVX infection caused a lowered accumulation of H2B transcripts and protein. Moreover, silencing of the H2B gene by VIGS caused an increase in the level of endogenous SA, and led to a decrease in the spread and titre of PVX in inoculated plants.

## Materials and Methods

### Plant Material and Agrobacterium Infiltration

Wild type *N. benthamiana* and *NahG* transgenic *N. benthamiana* plants (provided by Dr Yule Liu, Tsinghua University, Beijing, China) were grown under a 16-h day at 22°C and an 8-h night at 18°C. The Agrobacterium strain GV3101 was used and infiltration was performed as described (Liu et al., 2002). Equal volumes of individual agrobacterium cultures (OD_600_ = 1.0) were mixed before co-infiltration. GFP fluorescence was observed under long-wavelength UV-light (Black Ray model B 100A, Ultra-Violet Products Ltd., Upland, California, United States) and photographed using a Cannon digital camera.

### Virus-Induced Gene Silencing (VIGS)

The TRV vectors were kindly provided by Dr Yule Liu, Tsinghua University, Beijing, China (Liu et al., 2002). pTRV containing pYL279a TRV RNA2 was used to express the partial sequence of different plant genes in order to silence them. A fragment of about 300 bp of the N terminal of *N. benthamiana* H2B gene was inserted into the TRV RNA2 expression vector (creating TRV:H2B) and co-infiltrated into plants in combination with a TRV RNA1 vector as described before (Liu et al., 2002). A control infection consisted of the TRV RNA1 vector infiltrated in combination with an empty TRV2 vector (TRV:00), as previously described (Lu et al., 2016). Viral infection by Agrobacterium infiltration was performed as described (Jiang et al., 2014). The gene segments of *ICS* (EU257505.1), *EDS5* (Niben101Scf04767g00002.1) and *PAL2* (Niben101Scf05617g00005.1) were combined with the partial clones of H2B by overlap PCR to create a series of pTRV constructs for dual VIGS.

### Gene Expression Analysis

Total RNAs were isolated from TRV:00- and TRV:H2B-infected WT *N. benthamiana* and *NahG* transgenic plants with Trizol (Invitrogen, United States) according to the manufacturer’s instructions. The mRNA expression of H2B (EF189156) was analyzed by qRT-PCR. The *N. benthamiana* Ubiquitin C (UBC) gene (AB026056.1) was used as the internal reference gene for analysis (Rotenberg et al., 2006; Shi et al., 2016). A Roche LightCycler^®^480 Real-Time PCR System was used for the reaction and the results were analyzed by the ΔΔC_T_ method (Livak and Schmittgen, 2001). The primers used for qRT-PCR of SA pathway and silencing pathway-related genes are listed in Supplementary Table S1. The stability of the UBC reference gene as a baseline for relative quantitation calculations is shown in Supplementary Table S1.

### Western Blotting for Protein Detection

Histone proteins of plant samples were extracted with lysis buffer (10 mM Tris–HCl, pH 7.5; 2 mM EDTA; 0.25 M HCl; 5 mM DTT; 0.2 mM PMSF) as described (Qin et al., 2010). The histone protein pellet was dissolved with laemmli buffer (62.5 mM Tris–HCl, pH 6.8, 2% SDS, 25% glycerol, 0.01% bromophenol blue, and 10% β-mercaptoethanol), separated on 15% SDS–PAGE gel, detected with anti-H2B (Santa Cruz Biotechnology, Europe) and anti-actin (abbkine, Inc., WuHan, China) primary antibody and anti-rabbit (Sigma-Aldrich, St. Louis, MO, United States) secondary antibody. The antigen–antibody complexes were visualized using nitrotetrazolium blue chloride/5-bromo-4-chloro-3-indolyl phosphate (NBT/BCIP) buffer (Sigma-Aldrich, St. Louis, MO, United States) under standard conditions. Total proteins of plant samples were extracted with lysis buffer (100 mM Tris–HCl, pH 8.8, 60% SDS, 2% β-mercaptoethanol). Proteins were separated in a 12% SDS-PAGE gel and detected with anti-PVX CP primary antibody (Hangzhou Huaan Biotechnology Co., Ltd., (HuaBio)) and anti-rabbit (Sigma-Aldrich, St. Louis, MO, United States) secondary antibody. After incubation with secondary antibody, proteins were visualized with the EasySee Western Blot Kit (Transgene Biotech, BeiJing, China) and imaged with the Molecular Imager ChemiDoc Touch (Bio-Rad). Quantitative analysis of digital images of Western blots was done using ImageJ software.

### Detection of SA in Leaves by LC-MS

TRV-VIGS H2B-silenced and mock infiltrated leaves were harvested at 7 dpi. For SA quantification, approximately 50 mg leaf tissue was finely ground in liquid nitrogen and extracted with 400 μl of 10% methanol containing 1% acetic acid to which internal standards had been added (13.8 ng ^2^H_4_ SA). The quantification of SA was determined by LC-MS (Agilent 1260 Infinity-Agilent 6420A) as described previously (Forcat et al., 2008). Three independent replicates were performed with each experiment containing three biological repeats. The level of SA was measured by Zoonbio Biotechnology Co., Ltd.

## Results

### The Accumulation of H2B Was Reduced in PVX-Infected *N. benthamiana*

**FIGURE 1:**
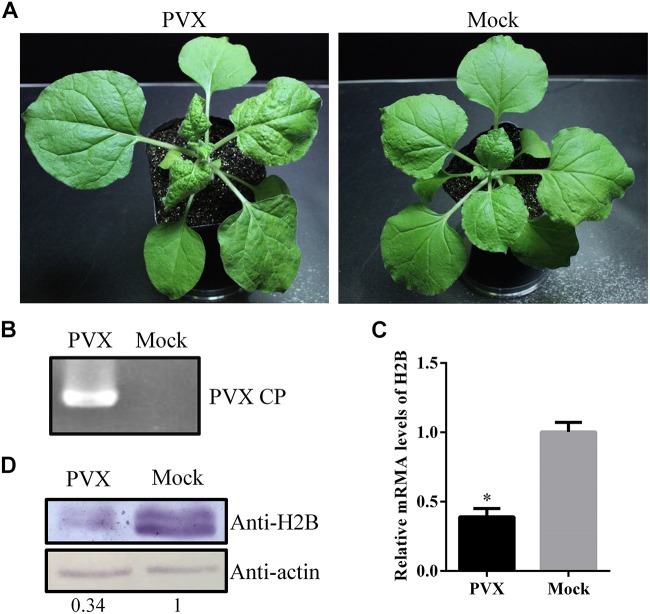
Expression levels of Histone H2B (H2B) transcripts and proteins in potato virus X (PVX)-infected *Nicotiana benthamiana*. **(A)** PVX symptoms at 6 dpi in *N. benthamiana* (left) and mock-inoculated plant (right). **(B)** PVX coat protein (CP) gene was detected by RT-PCR. **(C)** H2B proteins in PVX infected or healthy plants were detected by western blot using the H2B antibody. The relative amount of accumulated H2B protein was calculated using actin as a reference protein and is indicated below the panel. **(D)** After PVX infection, the expression of H2B transcripts was examined by qRT-PCR. Experiments were repeated three times. Bars represent the standard errors of the means. A two-sample unequal variance directional *t*-test was used to test the significance of the difference (^∗^*P* < 0.05).

*Nicotiana benthamiana* plants were inoculated with PVX mechanically. At 6 days post-inoculation (dpi), viral infection symptoms appeared on upper, uninoculated leaves of the PVX infected plants but not on the mock control plants (Figure 1A), and the viral infection was verified by RT-PCR with PVX CP gene-specific primers (Figure 1B). Quantitative reverse transcription (qRT)-PCR analysis at 6 dpi showed that the H2B mRNA levels in systemic PVX-infected leaves were only about 40% of those in uninfected control leaves (Figure 1C). Western blotting using an H2B-specific antibody showed that the H2B protein level was reduced in PVX-infected leaves to about 35% of that in the mock-inoculated controls (Figure 1D). However, at 13 dpi, a qRT-PCR assay showed that the expression level of H2B mRNAs in systemic leaves of PVX infected plants had recovered to about 70% of that in mock plants (Supplementary Figure S1).

### Abnormal Leaf and Stem Morphologies in VIGS-Treated H2B-Silenced Plants

**FIGURE 2:**
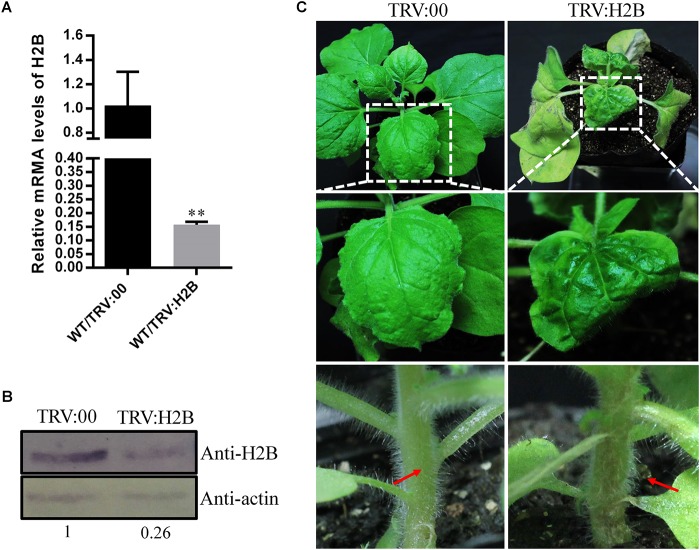
Effects of TRV-induced NbH2B silencing on *N. benthamiana* (WT) at 10 days dpi. **(A)** Validation of down-regulation of H2B transcripts in TRV:H2B *N. benthamiana* plants by qRT-PCR. Three repeat experiments were performed. Bars represent the standard errors of the means. A two-sample unequal variance directional *t*-test was used to test the significance of the difference (^∗∗^*P* < 0.01). **(B)** Western blot detection of H2B protein in H2B silenced (TRV:H2B) and non-silenced (TRV:00) plants. The relative amount of accumulated H2B protein was calculated using actin as a reference protein and is indicated below the panel. **(C)** H2B silencing caused the newly emerged leaves to be abnormal, yellow and curved (right upper and middle panels) compared with TRV:00 treated mock plants (left upper and middle panels) at 10 dpi. After treatment with TRV:H2B at 10 dpi, local necrosis emerged at the junction of the petioles (right bottom panel, red arrow) in H2B silenced plants, but there was no necrosis in the TRV control treated plants (left bottom panels, red arrow).

Since PVX infection resulted in reduced expression of H2B, we next investigated whether down-regulation of H2B expression using tobacco rattle virus (TRV)-induced gene silencing (VIGS) would have an effect on PVX infection. qRT-PCR confirmed that the TRV:H2B VIGS construct reduced the H2B transcript level by 85% compared with TRV:00-treated control plants at 10 dpi (Figure 2A). Moreover, the H2B protein concentration in silenced plants was only 26% of that in control plants (Figure 2B).

Leaf and stem malformations occurred on plants where the H2B gene was silenced by TRV VIGS, but not on TRV-treated control plants. Thus, at 10 dpi, malformed and chlorotic leaves developed, and petiole and main stem necrosis occurred on newly emerged leaves of plants agro-infiltrated with the TRV:H2B construct (Figure 2C), whereas there were no obvious symptoms on TRV:00 control plants (Figure 2C).

Semi-quantitative RT-PCR analysis showed that the levels of TRV CP were similar in both H2B-silenced and non-silenced control plants at 10 dpi (Supplementary Figure S2) suggesting that the chlorosis and necrosis of the leaves and stems was not caused by an increase in TRV titre in the silenced plants but was more likely an increased “sensitivity” of the plant to TRV infection.

### Silencing H2B Interfered With the Infection of *N. benthamiana* by PVX

**FIGURE 3:**
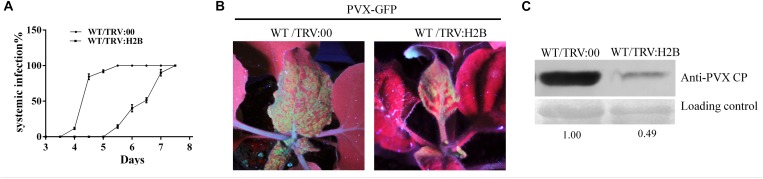
Decreased accumulation of PVX in H2B silenced WT plants. **(A)** Time-course results of PVX systemic infection index in H2B silenced and non-silenced WT plants. The proportion of systemically infected plants was determined by monitoring the green fluorescence on upper leaves under UV. The experiment was performed with 10 plants for each treatment and repeated three times. **(B)** Systemic PVX-GFP fluorescence at 7 dpi. The intensity of fluorescence on TRV:00 treated plants than on TRV:H2B treated plants. **(C)** Western blot detection of PVX CP protein at 7 dpi. The relative amount of accumulated PVX CP protein was calculated by comparing the CP band intensity with the loading control and is indicated below the panel.

At 10 dpi, after TRV mediated H2B-silencing system had been fully established in WT *N. benthamiana* plants, the plants were further challenged with a modified clone of PVX expressing the green fluorescent protein (PVX-GFP) and monitored for symptom development. Typical PVX symptoms of vein chlorosis and leaf curling appeared at 5 dpi on the top leaves of control (non-silenced) plants inoculated with PVX-GFP (Figure 3A). Newly emerging leaves show mosaic symptoms and green fluorescence under UV illumination (Figure 3B left). PVX became systemic 2 days later in the H2B-silenced plants (Figure 3A) and the green fluorescence appearing on most of the top leaves of H2B-silenced plants was weaker and the fluorescent area was less extensive than in the control plants (Figure 3B right). Western blotting showed that the PVX coat protein (PVX CP) accumulation in the systemic leaves was nearly 50% lower in H2B-silenced plants compared to the controls (Figure 3C).

### The Accumulation of Phytohormone SA Was Increased in H2B-Silenced *N. benthamiana* Plants

**FIGURE 4:**
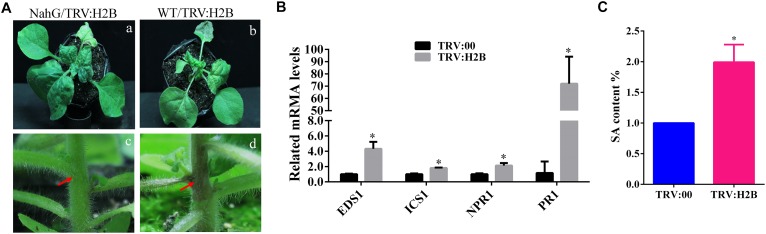
Salicylic acid (SA) accumulates in H2B silenced plant and is involved in petiole necrosis. **(A)** The symptoms caused by TRV:H2B VIGS on WT (panels **a,c**) or *NahG* (panels **b,d**) plants at 10 dpi. Local necrosis emerged at the junctions of stems and petioles (red arrows) in WT plants (panel **d**) but not *NahG* plants (panel **c**, red arrows). **(B)** qRT-PCR analysis of transcripts levels at 7 dpi of the EDS1, ICS1, NPR1, and PR1 genes in TRV:H2B WT plants and TRV:00 controls. A two-sample unequal variance directional *t*-test was used to test the significance of the difference (^∗^*P* < 0.05). **(C)** Relative levels of endogenous SA in TRV:00 and TRV:H2B treated WT plants as measured by LC-MS at 7 dpi. The endogenous SA in TRV:00 treated plant was set as the baseline. Three independent experiments were conducted with similar results.

One of the most well-known chemicals linked to the induction of necrosis in plants as a response to pathogen infection is SA (Alvarez, 2000; Brodersen et al., 2005; Dat et al., 2007; Straus et al., 2010). Transgenic plants expressing the bacterial enzyme salicylate hydroxylase (NahG), which degrades SA, have increased susceptibility to many plant pathogens, including viruses (Gaffney et al., 1993; Friedrich et al., 1995). We wondered whether the enhanced TRV symptoms seen in H2B-silenced plants could reflect changes to the SA synthesis and/or response pathway in these plants. We used *NahG* transgenic *N. benthamiana* plants, confirmed the expression of the transgene by RT-PCR with a specific primer pair (Supplementary Figure S3) and then also confirmed that TRV-VIGS was able to efficiently silence the H2B gene in these plants (Supplementary Figure S4). There was no obvious chlorosis on the newly emerging leaves and no necrosis of the petioles and stems of the *NahG* plants (Figure 4A) but these enhanced symptoms were produced in the non-transgenic control plants and also in H2B-silenced 16c plants (Voinnet et al., 2000) that were transformed with the GFP gene, which were used as a further control (Supplementary Figure S5).

We next performed qRT-PCR to examine the transcript levels of four SA pathway-related genes in H2B-silenced plants: *Enhanced disease susceptibility 1* (*EDS1*), *Isochorismate synthase* *1* (*ICS1*), *Non-expressor of Pathogenesis Related Genes 1* (*NPR1*), and *Pathogenesis-Related Protein 1* (*PR-1*). All four genes, *EDS1*, *ICS1*, *NPR1*, and *PR-1*, were significantly up-regulated in the leaves of H2B-silenced plants compared with the non-silenced controls by 4.4, 1.7, 2.4, and 130-fold, respectively (Figure 4B). To further determine whether the biosynthesis of SA is affected by the silencing of the H2B gene, we quantified the levels of SA in H2B-silenced and non-silenced plants using high-performance liquid chromatography tandem mass spectroscopy (LC-MS). Because of the transient nature of the increase in phytohormone concentrations following stress stimuli, experiments were repeated three times, with each study containing three biological replicates. There was a 2-fold increase in SA levels in H2B silenced plants (Figure 4C). Together, these results demonstrate that knock-down of H2B leads to the generation of higher levels of SA.

### Changes in the SA Pathway Are Linked to Petiole and Stem Necrosis During H2B VIGS

**FIGURE 5:**
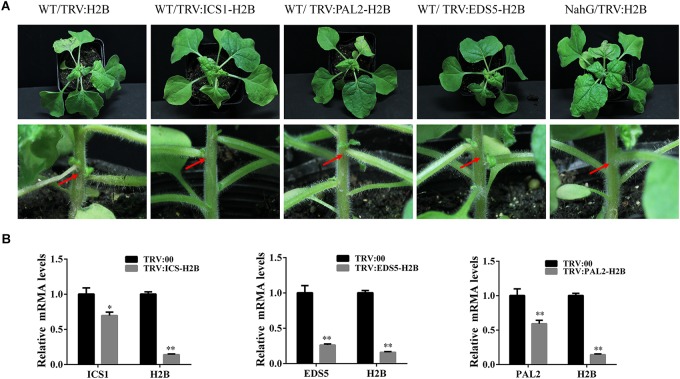
The symptoms of petiole and stem necrosis were compromised in TRV:ICS1-H2B, TRV:EDS5-H2B, and TRV:PAL2-H2B dual silenced plants. **(A)** Symptoms at 10 dpi caused by TRV mediated VIGS with TRV:ICS1-H2B, TRV:EDS5-H2B, and TRV:PAL2-H2B on WT and *NahG* plants. The presence of necrotic petioles (red arrow) was most apparent for the WT plant/TRV:H2B combination. TRV:ICS-H2B, TRV:EDS5-H2B, and TRV:PAL2-H2B had slight necrosis in stems on WT plants. There was almost no necrosis with the *NahG* plant/TRV:H2B combination. **(B)** Silencing of *H2B*, *ICS*, *EDS5*, and *PAL2* genes by the dual VIGS TRV constructs. Three repeat experiments were performed. Bars represent the standard errors of the means. A two-sample unequal variance directional *t*-test was used to test the significance of the difference (^∗^*P* < 0.05; ^∗∗^*P* < 0.01).

Necrosis is a hallmark of the hypersensitive reaction (HR) response that functions to combat virus infection in plants and is associated with SA synthesis. The absence of necrosis in the *NahG* plants adds further weight to the hypothesis that silencing of H2B alters the synthesis or turnover of SA. To further investigate the functioning of the SA pathway in H2B-silenced plants, we performed silencing experiments in which both H2B and other SA pathway-related genes were targeted in the same plant simultaneously. For this purpose, TRV2 VIGS constructs were made carrying both a fragment of H2B together with a fragment (300 bp) of either *ICS1*, *PAL2* (*phenylalanine ammonia-lyase-2*), or *EDS5*. Silencing of *ICS1*, *PAL2*, and *EDS5* genes was expected to reduce SA accumulation by targeting upstream steps in the SA synthesis pathway (Nawrath et al., 2002; Brodersen et al., 2005; Catinot et al., 2008). Leaves of plants infected with these constructs (TRV-ICS/H2B, TRV-PAL2/H2B, and TRV-EDS5/H2B) displayed foliar malformations similar to those that developed in plants infected with the original TRV-H2B construct at 10 dpi (Figure 5A, upper panels). However, these plants did not have petiole necrosis and so resembled *NahG* plants infiltrated with TRV-H2B rather than non-transgenic plants infiltrated with TRV-H2B (Figure 5A, bottom panels, red arrows). Silencing of the *ICS1*, *PAL2*, and *EDS5* genes in these plants was confirmed by qRT-PCR (Figure 5B). Expression of the *NPR1* gene, which is involved in downstream signaling in the innate immunity pathway, is upregulated by increases in the SA level (An and Mou, 2011). We therefore used qRT-PCR to examine the *NPR1* transcript levels in the dual-VIGS (TRV-ICS/H2B, TRV-PAL2/H2B, and TRV-EDS5/H2B) treated plants and found that *NPR1* expression was reduced in these plants but increased in H2B-silenced plants (Supplementary Figure S6).

Reducing either the synthesis of SA in dual VIGS-treated plants or the degradation of SA in *NahG* plants prevented the induction of petiole necrosis. These results corroborate the hypothesis that the petiole necrosis induced by H2B silencing is caused by activation of the SA pathway and the subsequent increase in SA content in these plants.

### Accumulated Endogenous SA in H2B-Silenced Plants Increases Transcription of Genes in Both the SA and RNA Silencing Pathways

**FIGURE 6:**
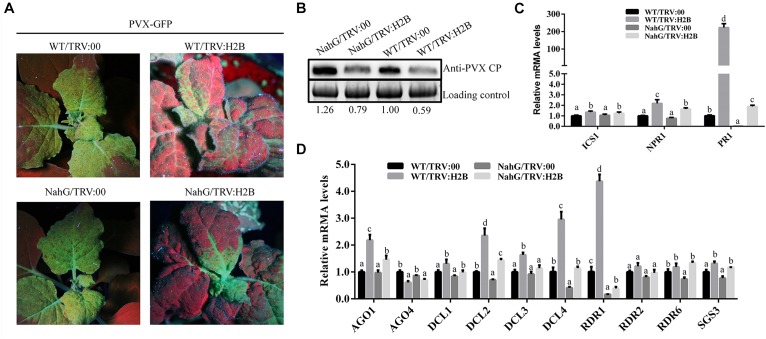
Expression of RNA silencing pathway genes was affected in H2B-silenced plants. **(A)** Systemic infection by PVX-GFP at 7 dpi on plants pre-treated with TRV:00 or TRV:H2B. **(B)** Western blot analysis of PVX CP protein in H2B silenced and TRV control treated WT and *NahG* plants. The level of PVX CP was calculated in relation to the loading control by comparing band intensities. **(C)** The relative expression levels of SA signaling pathways genes ICS1, NPR1 and PR1 in TRV:00 and TRV:H2B treated WT and *NahG* plants. **(D)** The relative expression levels of RNA silencing related genes *AGO1*, *AGO4*, *DCL1*, *DCL2*, *DCL3*, *DCL4*, *RDR1*, *RDR2*, *RDR6*, and *SGS3* in TRV:00 and TRV:H2B treated WT and *NahG* plants at 7 dpi. Error bars show SD and the graph represents the combined data from three independent replicates. Letters on the graph denote statistically significant differences (ANOVA, *P* ≤ 0.05).

Previous reports showed that treatment with SA induced resistance to PVX in *N. benthamiana* (Lee et al., 2011). We have shown here, that silencing H2B in non-transgenic *N. benthamiana* plants induced the accumulation of endogenous SA and decreased the level of PVX accumulation. In further experiments we found that PVX levels were higher in *NahG* plants compared to WT plants, and also higher in H2B-silenced *NahG* plants compared to H2B-silenced WT plants (Figure 6A,B). These results show that up-regulation of SA accumulation by H2B silencing does not completely overcome the SA degradation caused by the *NahG* transgene.

To further investigate this effect, the transcription levels of the SA synthesis gene *ICS1* and the SA responsive genes *NPR1* and *PR1* were analyzed by qRT-PCR in non-silenced WT, non-silenced *NahG* plants, H2B-silenced WT plants and H2B-silenced *NahG* plants. Small but significant increases in the *ICS1* transcript levels were seen when comparing H2B-silenced plants with non-silenced plants but there was no observable difference between the levels in the *NahG* and WT plants (Figure 6C). However, the transcription levels of the SA responsive genes *NPR1* and *PR1* were significantly decreased in H2B-silenced *NahG* plants compared with H2B-silenced WT plants (Figure 6C), reflecting the reduction of SA levels in *NahG* plants. In contrast, however, the transcription levels of *NPR1* and *PR1* were greatly up-regulated in H2B-silenced *NahG* plants compared with non-silenced *NahG* plants and in H2B-silenced WT plants compared to non-silenced WT plants (Figure 6C). These experiments show that silencing of the H2B gene increases expression of the SA responsive genes *NPR1* and *PR1*, and that the SA degradative activity of the *NahG* transgene is not sufficient to overcome this effect.

In tomato, SA induces expression of the RNA silencing-related genes *DCL2*, *RDR1*, and *RDR2*, thereby enhancing the resistance of the plant to the tomato mosaic virus (ToMV) (Campos et al., 2014). To investigate whether the increase in endogenous SA following H2B silencing in *N. benthamiana* similarly altered the expression of RNA silencing-related genes, we performed a qRT-PCR analysis of the transcription of the *N. benthamiana* homologs of *DICER 1* (*NbDCL1*), *DICER 2* (*NbDCL2*), *DICER 3* (*NbDCL3*), *DICER 4* (*NbDCL4*), *ARGONAUTE 1-1* (*NbAGO1-1*), *ARGONAUTE 4-1* (*NbAGO4-1*), *SGS3* (*NbSGS3*), *RDR1* (*NbRDR1)*, *RDR2* (*NbRDR2*), and *RDR6* (*NbRDR6*) (Li et al., 2014). As before, RNA samples were analyzed from H2B-silenced WT plants, non-silenced WT plants, H2B-silenced *NahG* plants and non-silenced *NahG* plants. For the non-transgenic, WT plants, silencing of the H2B gene by VIGS reproducibly and significantly increased the transcript levels of *NbAGO1-1*, *NbDCL2*, *NbDCL3*, *NbDCL4*, and *NbRDR1* by 2.5, 2.7, 1.6, 3.4, and 4.1 fold, respectively, whereas there were no significant changes in expression of the other genes (*AGO4*, *DCL1*, *RDR2*, *RDR6*, and *SGS3*) (Figure 6D). The level of *NbAGO1-1*, *NbDCL2*, *NbDCL3*, *NbDCL4*, and *NbRDR1* transcript increase was higher in WT plants as compared to *NahG* plants by 1.4, 2, 1.5, 2.7, and 2.2 fold, respectively, suggesting that, as before, it is the increase in endogenous SA level that leads to the increase in expression of these RNA silencing-related genes. In *NahG* plants the absolute level of gene expression fold-change was reduced compared to non-transgenic plants but H2B silencing still resulted in significant increase in expression of *AGO1*, *DCL2*, *DCL4*, and *RDR1*.

## Discussion

Although H2B histone is known to be involved in plant defense responses against fungi, no studies linking H2B and plant viruses have yet been reported (Hu et al., 2014; Li et al., 2015; Zhang et al., 2015). Here, we show that the expression level of the H2B mRNA and the accumulation of the H2B protein in *N. benthamiana* is decreased during infection by the RNA virus PVX. Knock down of H2B expression in *N. benthamiana*, also led to the abnormal development of leaves and the induction of petiole and stem necrosis. However, PVX infection, caused low expression of H2B, and resulted in milder symptoms than in H2B silenced plants (Figure 1A, 2C). We presume that the mild symptoms are due to the recovery in expression levels of H2B in PVX infected plants at 11 and 13 dpi (Supplementary Figure S1). We also found that artificially reducing H2B expression by TRV-mediated VIGS leads to a reduction in the accumulation of PVX in the plants, which was related to the induction of endogenous SA.

Deficiencies in nuclear lamina proteins CROWDED NUCLEI (CRWN) induces plant dwarfing and spontaneous cell death lesions, which are caused by over-production of SA in mutants (Choi et al., 2019). We hypothesized that the petiole necrosis in H2B silenced plants could be associated with changes in the production and/or accumulation of SA. We confirmed by LC-MS that the SA level is two-fold higher in H2B-silenced plants, and we further showed that transcription of a selection of SA pathway-related genes was up-regulated in these plants. By repeating the experiments in transgenic plants expressing the *NahG* gene we were able to confirm that reduction in the accumulation of SA by *NahG* enzyme activity inhibited the induction of petiole necrosis in H2B-silenced plants and also reduced the extent of the SA-related gene expression increase caused by the H2B silencing. A similar phenomenon was also observed for the *acd11* mutant of Arabidopsis, which has constitutive activation of PCD and other genes involved in defense against pathogens such that this mutation is lethal during early plant growth (Brodersen et al., 2005). However, combining the *acd11* mutation with the *NahG* transgene prevented the initiation of PCD and rescued the plant. It is also clear from this and other studies that the synthesis/accumulation of SA and the expression of various SA pathway genes are co-regulated, with both positive and negative feedback being identified (Shah, 2003; Loake and Grant, 2007; Chen et al., 2009; Vlot et al., 2009).

Whether the changes we observed in SA production and SA pathway gene expression are a direct or an indirect consequence of the silencing of the H2B gene, remains to be investigated. It would seem very possible that transcription of (some) SA pathway genes may be controlled directly by the binding of H2B to their promoters or by repressing the expression of other transcription factors that themselves bind to the SA pathway gene promoters.

The mechanism by which PVX infection affects the H2B level is not known. However, it was reported that histone H2B is strongly decreased in response to DNA damage (ionizing radiation) through modulation of octamer transcription factor 1 (Schild et al., 2003). Several reports indicate that virus infection, including plant viruses, can induce DNA damage (Gruhne et al., 2009; Pal et al., 2010; Cerovska et al., 2014). Particularly, there is evidence that PVX induces DNA damage in nuclei isolated from tobacco leaves (Cerovska et al., 2014). Despite being a positive-strand RNA virus that replicates in the cell cytoplasm, some viral proteins encoded by PVX can localize to the nucleus (Samuels et al., 2007). Furthermore, it has been found that SA-mediated defense gene expression is up-regulated by DNA-damaging agents and by mutation in DNA damage repair processes, which could be linked to H2B activity (Yan et al., 2013).

Finally, we showed that the expression of the RNA silencing related genes *NbAGO1-1*, *NbDCL2*, *NbDCL3*, *NbDCL4*, and *NbRDR1* is up-regulated in H2B-silenced plants. We expect that these changes in gene expression are initiated by the increase in SA following H2B silencing. The tobacco *RNA-dependent RNA polymerase 1* (*RdRP1*) gene, that functions during RNA silencing to amplify target dsRNAs, was found to be up-regulated by both TMV and SA treatment (Xie et al., 2001). More recently, in tomatoes, SA treatment was shown to up-regulate the expression of *DCL1*, *DCL2*, *RDR1*, *RDR2*, and repress the expression of DCL4 and RDR6 (Campos et al., 2014). Thus, it is becoming increasingly clear that the actions of the SA pathway and the RNA silencing pathway as a defense against viruses are coordinated in plants. In addition, other plant hormones such as jasmonic acid (JA), abscisic acid (ABA) and auxin are reported to be involved in crosstalk with SA (An and Mou, 2011; Proietti et al., 2013; Munoz-Espinoza et al., 2015), although we have not extended the work in our study to include these signaling pathways. Further work will be required to understand the precise mechanism(s) by which H2B, and perhaps other histone proteins or plant hormones, are integrated into plant defense pathways.

## Data Availability

The raw data supporting the conclusions of this manuscript will be made available by the authors, without undue reservation, to any qualified researcher.

## Author Contributions

XY, YL, JC, and FY designed the experiments. XY, YL, XZ, LJ, SX, JP, HZ, LL, and YW performed the experiments and interpreted the data. YL and FY drafted the manuscript. SM and JC revised the manuscript.

## Conflict of Interest Statement

The authors declare that the research was conducted in the absence of any commercial or financial relationships that could be construed as a potential conflict of interest.

## Supplementary Material

FIGURE S1 Time course of expression levels of H2B transcripts in PVX infected systemic leaves. qRT-PCR analysis of the expression levels of H2B transcripts in PVX infected systemic leaves at 7, 9, 11, and 13 dpi.

FIGURE S2 Levels of TRV are not altered by H2B silencing. The accumulation of the TRV CP gene in TRV:00 and TRV:H2B treated plants was analyzed by semi-quantitative RT-PCR at 10 dpi.

FIGURE S3 *NahG* transgenic plant validation by RT-PCR. Expression of the *NahG* gene in transgenic plants was confirmed by RT-PCR in *NahG*/TRV:00 and *NahG*/TRV:H2B plants. No *NahG* amplification occurred in WT plants.

FIGURE S4 Validation of H2B down regulation in TRV:H2B treated *NahG* plants. The expression of H2B was down-regulated by about 75% in TRV:H2B treated *NahG* plants. Three repeat qRT-PCR experiments were performed. Bars represent the standard errors of the means. A two-sample unequal variance directional *t*-test was used to test the significance of the difference (^∗∗^*P* < 0.01).

FIGURE S5 Phenotype and petiole necrosis on H2B silenced and non-silenced WT, *NahG*, and 16C plants. There were no observed symptoms on non-silenced WT, *NahG*, and 16C plants. Abnormal foliar developments were seen on H2B silenced WT, *NahG*, and 16C plants. Additionally, petiole necrosis occurred on H2B silenced WT and 16C plants but not on *NahG* plants (red arrow).

FIGURE S6 The NPR1 transcript level in TRV:00, TRV:H2B and three dual VIGS plants. The transcript level of NPR1 in TRV:ICS1-H2B, TRV:EDS5-H2B, and TRV:PAL2-H2B treated plants was significantly down-regulated compared with TRV:00 and TRV:H2B treated plants. Error bars show SD and the graph represents the combined data from three independent replicates. Letters on the graph denote statistically significant differences (ANOVA, *P* ≤ 0.05).

TABLE S1 Primer pairs used in this study.
