# Supplementary figures and images for: Downregulation of Nuclear Protein H2B Induces Salicylic Acid Mediated Defense Against PVX Infection in Nicotiana benthamiana
Source: Front Microbiol. 2019 May 8;10:1000. doi: 10.3389/fmicb.2019.01000 (PMC6517552; doi:10.3389/fmicb.2019.01000)

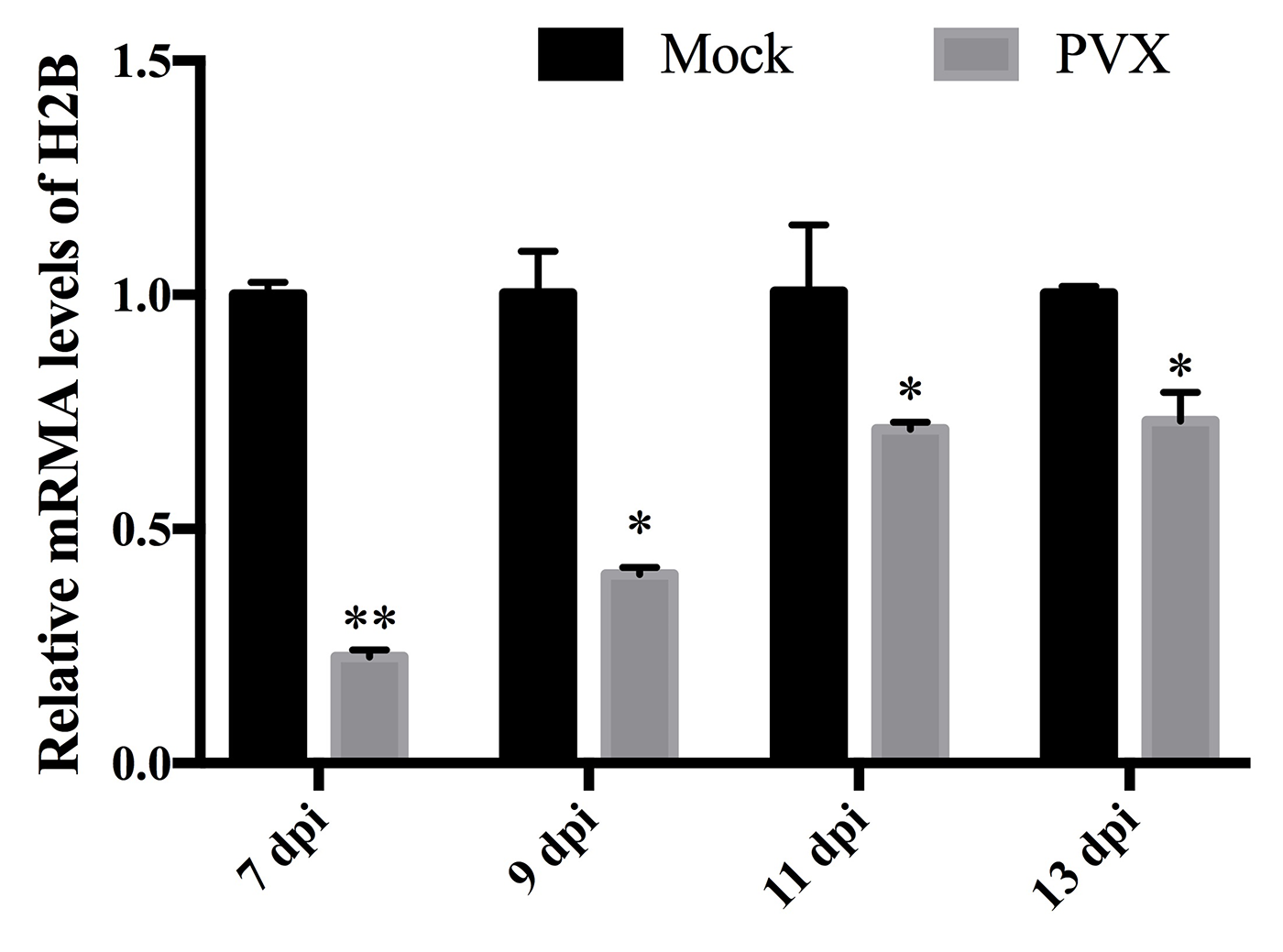

Supplement: FIGURE S1 — Time course of expression levels of H2B transcripts in PVX infected systemic leaves. qRT-PCR analysis of the expression levels of H2B transcripts in PVX infected systemic leaves at 7, 9, 11, and 13 dpi. [file Image_1.TIF]

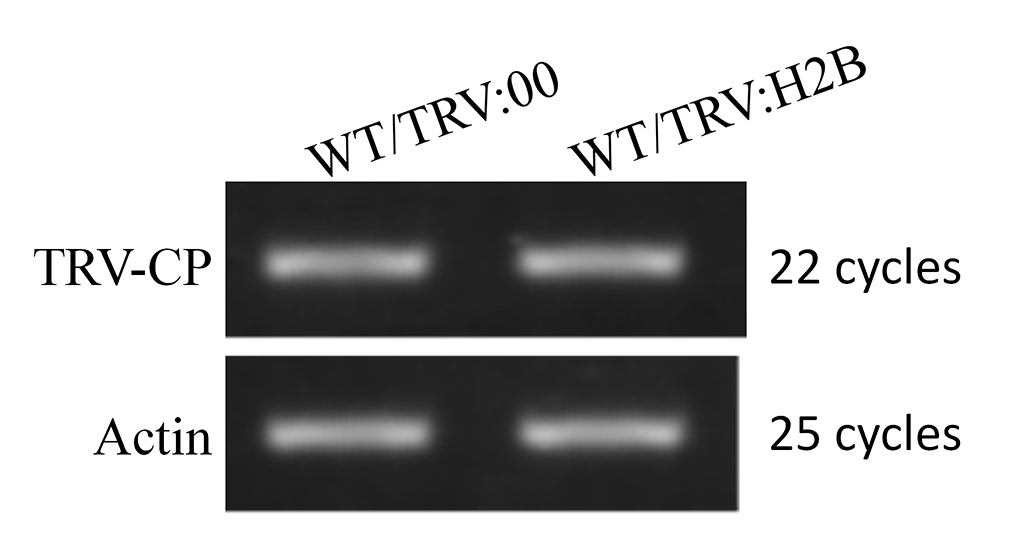

Supplement: FIGURE S2 — Levels of TRV are not altered by H2B silencing. The accumulation of the TRV CP gene in TRV:00 and TRV:H2B treated plants was analyzed by semi-quantitative RT-PCR at 10 dpi. [file Image_2.TIF]

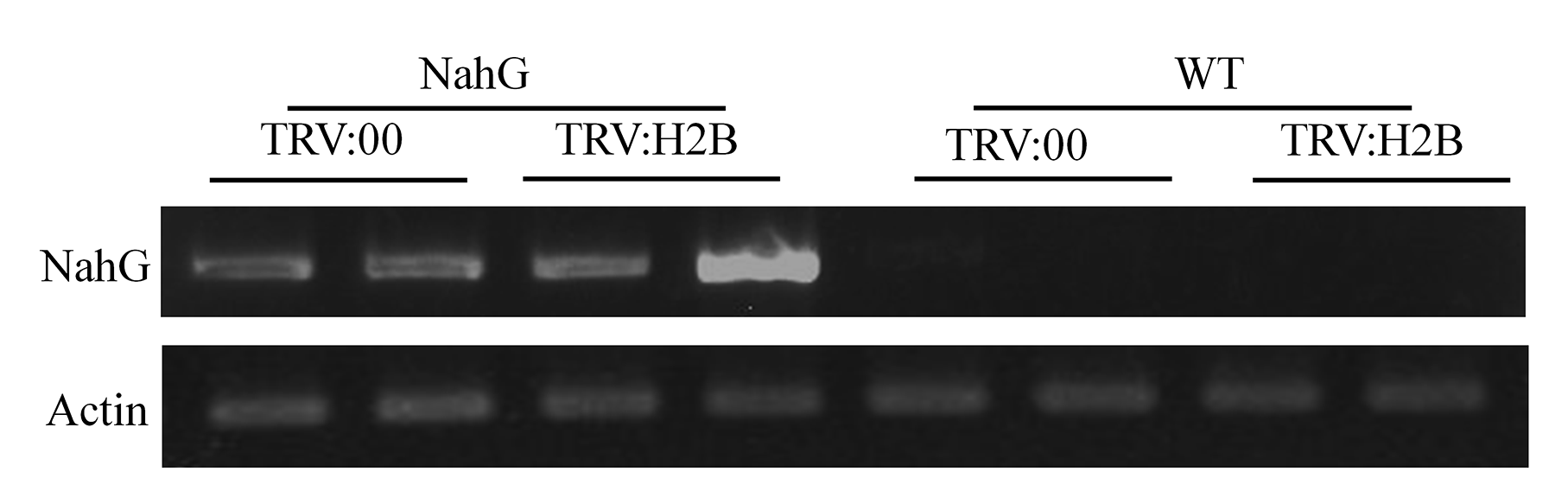

Supplement: FIGURE S3 — NahG transgenic plant validation by RT-PCR. Expression of the NahG gene in transgenic plants was confirmed by RT-PCR in NahG/TRV:00 and NahG/TRV:H2B plants. No NahG amplification occurred in WT plants. [file Image_3.TIF]

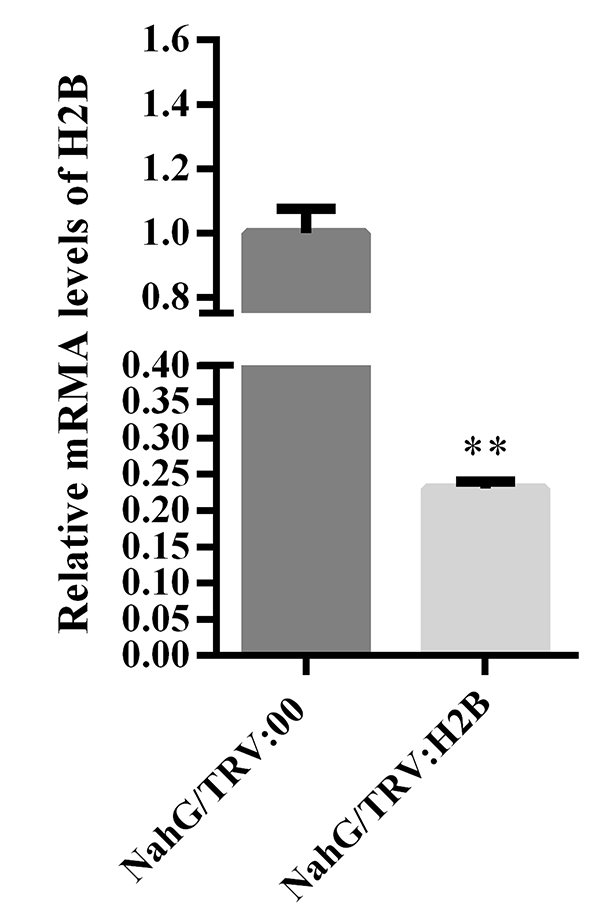

Supplement: FIGURE S4 — Validation of H2B down regulation in TRV:H2B treated NahG plants. The expression of H2B was down-regulated by about 75% in TRV:H2B treated NahG plants. Three repeat qRT-PCR experiments were performed. Bars represent the standard errors of the means. A two-sample unequal variance directional t-test was used to test the significance of the difference (∗∗P < 0.01). [file Image_4.TIF]

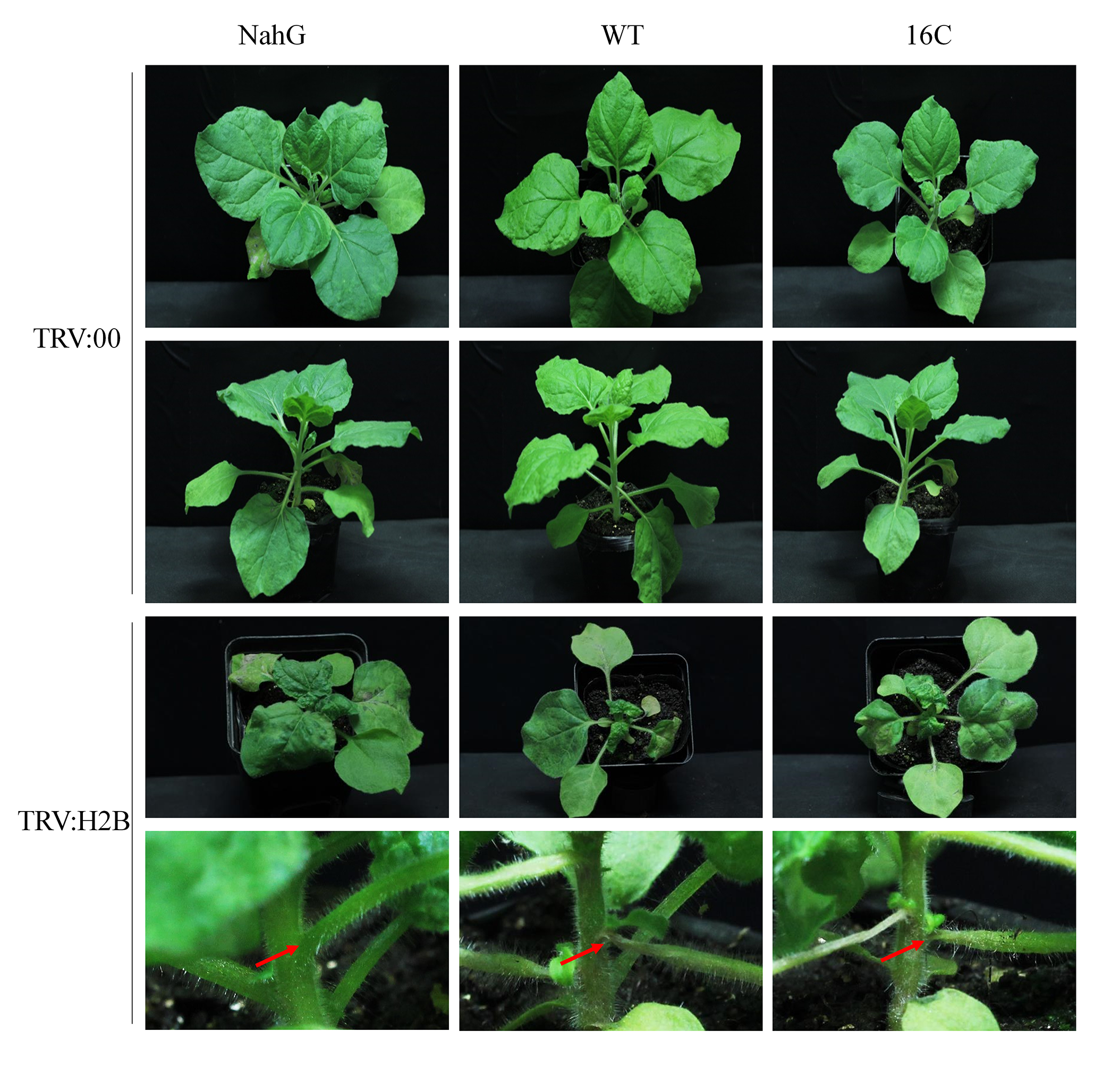

Supplement: FIGURE S5 — Phenotype and petiole necrosis on H2B silenced and non-silenced WT, NahG, and 16C plants. There were no observed symptoms on non-silenced WT, NahG, and 16C plants. Abnormal foliar developments were seen on H2B silenced WT, NahG, and 16C plants. Additionally, petiole necrosis occurred on H2B silenced WT and 16C plants but not on NahG plants (red arrow). [file Image_5.TIF]

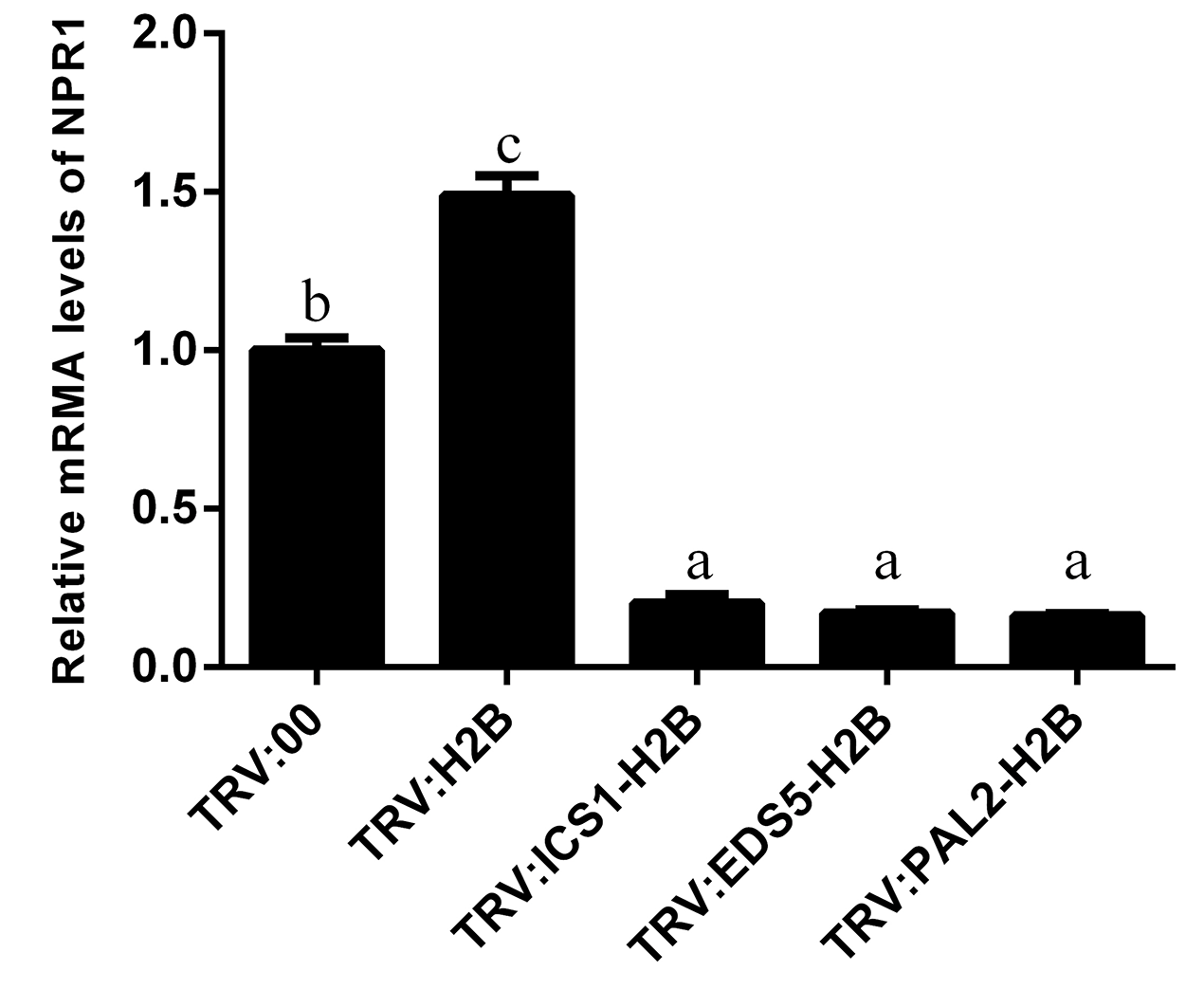

Supplement: FIGURE S6 — The NPR1 transcript level in TRV:00, TRV:H2B and three dual VIGS plants. The transcript level of NPR1 in TRV:ICS1-H2B, TRV:EDS5-H2B, and TRV:PAL2-H2B treated plants was significantly down-regulated compared with TRV:00 and TRV:H2B treated plants. Error bars show SD and the graph represents the combined data from three independent replicates. Letters on the graph denote statistically significant differences (ANOVA, P ≤ 0.05). [file Image_6.TIF]
