# Supplementary material for: Downregulation of Nuclear Protein H2B Induces Salicylic Acid Mediated Defense Against PVX Infection in Nicotiana benthamiana
Source: Front Microbiol. 2019 May 8;10:1000. doi: 10.3389/fmicb.2019.01000 (PMC6517552; doi:10.3389/fmicb.2019.01000)
Supplement: TABLE S1 — Primer pairs used in this study. [file Table_1.DOC]

**Supplementary Table 1: Primers used in the study**

| Primers | | Use |
| --- | --- | --- |
| NBH2B VIGS-F | 5'-AAAACCACCGTCGCCGAGAA-3' | VIGS |
| NBH2B VIGS-R | 5'- CTTGCTCGAAATCCCAATGT -3' | VIGS |
| NBH2B RT-F | 5'- GCCCACAATCACTTCTCG-3' | qRT-PCR |
| NBH2B RT-R | 5'-TGAACTTGGTAACAGCCTT-3' | qRT-PCR |
| NBUBC RT-F | 5'-GAGGAAGAGACTGGTGAGGGAT-3' | qRT-PCR |
| NBUBC RT-R | 5'-CACAGAGCAAAGACTGGATTGA-3' | qRT-PCR |
| PVX CP F | 5'-ATGTCAGCACCAGCTAGCAC-3' | RT-PCR |
| PVX CP R | 5'-TGGTGGTGGG AGAGTGACAA-3' | RT-PCR |
| NAHG F | 5'-ATGAAAAACAATAAACTTGG-3' | RT-PCR |
| NAHG R | 5'-CACCCTTGAC GTAGCGCACC-3' | RT-PCR |
| NBEDS1 RT-F | 5'-CTGGGCGATGATGTTTTCTT-3' | qRT-PCR |
| NBEDS1 RT-R | 5'-CTAGGAGTACCCGCAAGCTG-3' | qRT-PCR |
| NBICS1 RT-F | 5'-GCAACAGCCAACATAGGTC-3' | qRT-PCR |
| NBICS1 RT-R | 5'-TCATAAGAACGGAGGAAACC-3' | qRT-PCR |
| NBNPR1 RT-F | 5'-TGAGATTCTGGAGCAAGCA-3' | qRT-PCR |
| NBNPR1 RT-R | 5'-GTTGTCCTCTGTGCGTTGA-3' | qRT-PCR |
| NBPR1A RT-F | 5'-TGGATTGTTCTGCTTGATATCA-3' | qRT-PCR |
| NBPR1A RT-R | 5'-TTGTTCCTAAGGCATGTAGTTAA-3' | qRT-PCR |
| NBAGO1 RT-F | 5'-CTGGCGTGGCTTCTATCAAAGTATT-3' | qRT-PCR |
| NBAGO1 RT-R | 5'-CACCTTTACACCTCTCAGTGCCTTC-3' | qRT-PCR |
| NBAGO4 RT-F | 5'-GGAACTATGACTTCTACCTGTGTGCC-3' | qRT-PCR |
| NBAGO4 RT-R | 5'-AACTTCATCCATTGTCCAACTTGTGT-3' | qRT-PCR |
| NBDCL1 RT-F | 5'-TCTTTACATACACAGATCTCCCC-3' | qRT-PCR |
| NBDCL1 RT-R | 5'-ACCTGGTTTTGATAGTTCATTTT-3' | qRT-PCR |
| NBDCL2 RT-F | 5'-GAAGGCAGAAGACTAAAAGTAAGAGG-3' | qRT-PCR |
| NBDCL2 RT-R | 5'- CTACAAGCAGAGAAGGATCATGGAAC-3' | qRT-PCR |
| NBDCL3 RT-F | 5'-GAGTCAACAGAGCGTAAATCCAAGTC-3' | qRT-PCR |
| NBDCL3 RT-R | 5'-CTGTCGTTCTAGCTCATACAGCATGA-3' | qRT-PCR |
| NBDCL4 RT-F | 5'- GCAAAGACTGAAGTTAGGTGTAGCAA-3' | qRT-PCR |
| NBDCL4 RT-R | 5'-TTTCATCAGCAAGTGGAATAAAGATT-3' | qRT-PCR |
| NBSGS3 RT-F | 5'-GTTCCTCCTGCTCTGAAGAATGG-3' | qRT-PCR |
| NBSGS3 RT-R | 5'-GGGCAGTGCCACTGTCGTTCAGG-3' | qRT-PCR |
| NBRDR1 RT-F | 5'-GACAACTTCCAAAATCTCCATCCA-3' | qRT-PCR |
| NBRDR1 RT-R | 5'-TTCTCCCACTCCTCATCAACAAAA-3' | qRT-PCR |
| NBRDR2 RT-F | 5'-GGCACCAACTCATTCTAACCTC-3' | qRT-PCR |
| NBRDR2 RT-R | 5'-CCAACCTATAACACTCCCCCTT-3' | qRT-PCR |
| NBRDR6 RT-F | 5'-GTATGCGGAACTTGAATAGGAATGTG-3' | qRT-PCR |
| NBRDR6 RT-R | 5'-GGTCTTCTGCAAAAAACCAGGCAGA-3' | qRT-PCR |
| ICS RT-F | 5'-TCGTCCATTCACCCTACTCC-3' | qRT-PCR |
| ICS RT-R | 5'-CCAAGACCCTTTTCAACCAA-3' | qRT-PCR |
| EDS5 RT-F | 5'-TCCATGGTTATTCCCCAAAA-3' | qRT-PCR |
| EDS5 RT-R | 5'-AGGTAAGGCGTCGTAGAGCA-3' | qRT-PCR |
| PAL2 RT-F | 5'-GATTGGAGCTTTCGAAGACG-3' | qRT-PCR |
| PAL2 RT-R | 5'-CGGTGATCGGACTCTTTCTC-3' | qRT-PCR |
| ICS F | 5'-AAAAAGCAGGCTCCTGCTGCAACTATTGCATGGG-3' | VIGS |
| ICS R | 5'-AGAAAGCTGGGTCCCCACAAACTGCTGGAGTAG G-3' | VIGS |
| EDS5 F | 5'-AAAAAGCAGGCTCCGTCTCGATTTTTTATTGCTC-3' | VIGS |
| EDS5 R | 5'-AGAAAGCTGGGTCGAGCAATAAAAAATCGAGAC-3' | VIGS |
| PAL2 F | 5'-AAAAAGCAGGCTCCCTAATGCAAAAACTGAGACA-3' | VIGS |
| PAL2 R | 5'-AGAAAGCTGGGTCTGTCTCAGTTTTTGCATTAG-3' | VIGS |
| ICS-H2B LF | 5'-CCTACTCCAGCAGTTTGTGGGTAA  GCACCAAAAGCCGAGAA-3' | Double VIGS |
| ICS-H2B LR | 5'-TTCTCGGCTTTTGGTGCTTACCCA  CAAACTGCTGGAGTAGG-3' | Double VIGS |
| EDS5-H2B LF | 5'-CCCGAAAAATGAACTGAATCCAAT  AAGCACCAAAAGCCGAGAA-3' | Double VIGS |
| EDS5-H2B LR | 5'-TCTCGGCTTTTGGTGCTTATTGGATT  CAGTTCATTTTTCGGG-3' | Double VIGS |
| PAL2-H2B LF | 5'-ATCCAATGCTGGAGTGTCTCTAA  GCACCAAAAGCCGAGAA-3' | Double VIGS |
| PAL2-H2B LR | 5'-TTCTCGGCTTTTGGTGCTTAGAGA  CACTCCAGCATTGGAT-3' | Double VIGS |
